# Palm oil based stretchable piezoresistive strain sensors

**DOI:** 10.1016/j.heliyon.2024.e40791

**Published:** 2024-11-28

**Authors:** Vani Virdyawan, Thoriq Marendra, Bagas Prakoso, Indrawanto, Afriyanti Sumboja

**Affiliations:** a Engineering Design and Production Research Group, Faculty of Mechanical and Aerospace Engineering, Institut Teknologi Bandung, Jl. Ganesa 10, Bandung, 40132, Indonesia; b Material Science and Engineering Research Group, Faculty of Mechanical and Aerospace Engineering, Institut Teknologi Bandung, Jl. Ganesa 10, Bandung, 40132, Indonesia; c Mekanisasi Perikanan, Politeknik Kelautan dan Perikanan Sorong, Jl. Kapitan Pattimura, Sorong, 98411, Indonesia

**Keywords:** Organic solvent, Carbon black, Flexible sensor, Human motion detection, Soft sensor, Soft robots

## Abstract

The advancement of wearable devices and soft robots requires soft and stretchable sensors to detect their movements. This article proposes palm oil as an organic solvent for a stretchable piezoresistive strain sensor made from a composite consisting of elastomer (Ecoflex 00–30) filled with carbon black. The high content of palmitic acid in the palm oil increases the dispersity of carbon black in the composite, hence effectively improving the conductivity of the sensors. Furthermore, using palm oil as a natural plasticizer can lower the degree of crosslinking of the matrix, reducing the modulus elasticity but still producing a stretchable sensor with 500 % elongation at break. The presence of palm oil in the sensor also increases the gauge factor, showing a value of 2.43–4.75 and better repeatability during loading. These gauge factors are associated with two linear strain regions of the sensors (R^2^ > 0.99), which are 20–200 % and 0–20 % strain, respectively. The stretchable sensor also shows high durability that can withstand >1500 cycles at 60 % strain. The as-fabricated sensor can be deployed to detect the movement of the human body, such as for measuring a finger's joint angle and in soft robotics applications.

## Highlights

• Piezoresistive strain sensor is made using palm oil as a biocompatible solvent.
• Palm oil ensures the strain sensor has good conductivity and stretchability.
• The strain sensor offers up to 500 % strain and a resistance value of 21.54 kΩ.
• The sensor has a gauge factor of 2.43–4.75 and endures 1500 cycles at 60 % strain.
• The sensor is suitable for detecting body movements and soft robotics applications.

## Graphical abstract

**Figure:**
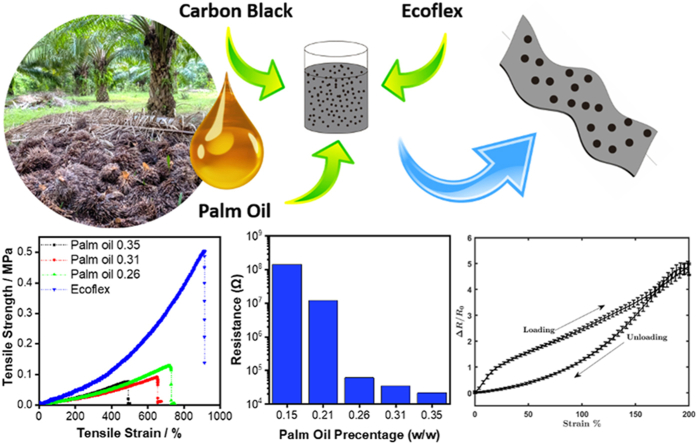

## 1 Introduction

Stretchable strain sensors are widely used to convert mechanical deformations into electrical signals in soft robotics, human-machine interaction, and health monitoring to provide greater accuracy, efficiency, and safety [[1], [2], [3], [4]]. Compared to their rigid counterparts, which can only be stretched <5 %, stretchable strain sensors can be extended for more than 100 % [5]. Based on their working principle, stretchable sensors can be classified into piezoresistive, piezocapacitive, piezoelectric, optical, and triboelectric [6]. Among them, the piezoresistive sensor is the most widely developed due to its simple structure, ease of fabrication, high sensitivity, good strain ability, and wide range of applications [1,5,[7], [8], [9]].

The working principle of piezoresistive sensors is based on the changes in the sensor dimension during loading, which results in a variation in its electrical resistance [7]. They are of interest for wearable electronics to serve as strain sensors in human-machine interfaces. For this application, a piezoresistive strain sensor ideally fulfils several requirements, including a high gauge factor, high elasticity, high flexibility, and wide detection range [7,10,11]. Piezoresistive strain sensors based on composite materials have been proposed to fulfil those requirements. By combining two or more materials with different properties, sensors with tailored properties, such as improved sensitivity, increased linearity, enhanced durability, and stretchability, can be obtained [[12], [13], [14], [15]]. However, due to the large search space, determining suitable material formulations and fabrication methods to produce affordable, high-performance, stretchable piezoresistive strain sensors is still an open challenge.

Stretchable piezoresistive strain sensors based on composite material may consist of matrix and conductive fillers. Ecoflex™, Dragonskin™, butadiene nitrile rubber, and styrene butadiene have been used as matrices in composite-based strain sensors due to their mouldability, processability, biocompatibility, and good mechanical properties [1,7]. Conductive fillers, such as silver and carbon-based materials, are then introduced to enhance the conductivity of the matrix [1,5,16,17]. Among conductive filler materials, carbon black is of interest due to its excellent electrical conductivity, high surface area, and low cost [5,7,17]. However, the dispersibility of carbon black in the matrix is pretty low. They are easy to agglomerate during the fabrication of the sensors, resulting in the difficulty of forming a consistent conductive network, which is required during the operation of resistive strain sensors [1,7,18].

Adding solvents to boost the dispersibility of carbon black becomes necessary in order to prevent the agglomeration of carbon black within the matrix. Chloroform [19], toluene [18], heptane [20], hexane, and acetone [21] have been used as a solvent to disperse carbon black. However, most of them are toxic or explosive, requiring extra care during fabrication [1]. Silicone oil has also been proposed as a non-toxic solvent to improve the dispersion of carbon black. However, it suffers from a limited stretchability of less than 170 % [22]. A non-toxic organic solvent, such as virgin coconut oil, has also been used to increase the dispersion of carbon black in the Ecoflex matrix [1]. The use of organic solvent produces a stretchable piezoresistive sensor with a porous structure filled with oily suspensions. The presence of the oily suspension in the sensor improves its conductivity and repeatability by reducing the friction between carbon black particles and the elastomer molecules. However, the sensor has a gauge factor of less than 1 (0.21–0.77) [1].

Even though promising, the use of an organic solvent to improve carbon black dispersion is still lacking in the literature. In this research, we investigate the use of palm oil as an organic solvent to improve the dispersion of carbon black in an elastomer matrix. While both coconut oil and palm oil are composed of triglyceride groups and palmitic acid [[23], [24], [25]], palm oil consists of more palmitic acid than coconut oil [[26], [27], [28]]. Palmitic acid is an amphipathic molecule with a hydrophilic headgroup that will induce electrostatic repulsion between carbon black, hence preventing agglomeration of the carbon black particles and enhancing conductivity for the sensor [29]. Besides that, palm oil is also a natural plasticizer, which may reduce the composite material's modulus elasticity, which is desirable for detecting human movement and soft robot applications. In addition, compared to virgin coconut oil, palm oil is available in larger quantities at a more affordable price [30].

To the best of our knowledge, a strain sensor based on palm oil solvent has yet to be reported in the literature. In this research, a stretchable piezoresistive strain sensor is synthesized using palm oil as a dispersing agent and natural plasticizer for the composite materials. The amount of palm oil was varied to evaluate the contribution of palm oil to the performance of the piezoresistive strain sensor. This study found that the more palm oil was added, the conductivity and gauge factor of the resulting sensor increased, and the repeatability also increased. The produced sensor can maintain its resistance value after being stretched and released for 1500 cycles at 60 % strain. Moreover, the sensor can be stretched up to 500 % and could be used to detect finger joint bending.

## 2 Experimental method

### 2.1 Fabrication of piezoresistive strain sensor

Carbon black (VULCAN XC72R, FuelCell Store, USA) with an average particle size of 50 nm and bulk density of 96 kg m^−3^ was employed as a conductive filler. Ecoflex™ 00–30 (Smooth-On, Inc., PA, USA) was used as a matrix with a viscosity of 3000 cps. Palm oil as an organic solvent was purchased from Indonesia's Convenient Store.

Ecoflex™ 00–30 part B, the carbon black, and the palm oil were mixed using a vacuum mixer (TOB, China). The mixture was stirred for 3 h at 350 rpm to ensure its uniformity. The mixture was then added with Ecoflex™ 00–30 Part A, which was mixed for 4 min to avoid curing during the mixing process. The composite material was then vacuumed for 15–20 min to remove air bubbles. The produced solution was then poured into a mould with a dimension of 20 × 5 × 2 mm (length × width × thickness). Another vacuum process was then carried out for 5–10 min to remove the air bubbles generated during the pouring. The amounts of Ecoflex Part A, Ecoflex Part B, Carbon Black, and palm oil in each sample were summarized in Table 1.

**Table 1:** The mass ratio between Ecoflex Part A: Ecoflex Part B: Carbon Black: Palm Oil.

Sample Variance	Ecoflex Part A/Ecoflex Part B	Carbon Black	Palm oil	Palm oil (w/w)
Palm Oil 0.15	2.5/2.5	0.6	1.0	0.15
Palm Oil 0.21	2.5/2.5	0.6	1.5	0.21
Palm Oil 0.26	2.5/2.5	0.6	2.0	0.26
Palm Oil 0.31	2.5/2.5	0.6	2.5	0.31
Palm Oil 0.35	2.5/2.5	0.6	3.0	0.35

### 2.2 Material characterizations and sensing performance

Qualitative characterizations of the sensor materials were performed using Fourier-transform infrared spectroscopy (FTIR, Bruker, Alpha II, Massachussets, USA) and Scanning electron microscopy (SEM, Hitachi SU 3500, Hitachi, Tokyo). The sensor's electrical resistance was measured using a precision digital multimeter (Fluke 8845A, Fluke Corporation, Washington, USA). The resistivity of the sensor was then calculated from these resistance measurements and the sensors' dimensions. Dogbone samples were prepared for each sample composition. A tensile strength test was performed using a universal testing machine (Instron 6800 Series, Illinois Tools Work, USA).

The resistance of the sensors was measured during loading (stretching) and unloading (releasing). The real-time change of the electrical resistance was recorded using a precision digital multimeter (Fluke 8845a, Fluke Corporation, Washington, USA) to derive the gauge factor and hysteresis of the sensors. To perform the loading and unloading cycles, the sample was clamped on top of a precision linear stage (X-LSME050A, Zaber, British Columbia, Canada). The gauge factor (GF) was used to describe the sensitivity of the sensor, which is defined as the change in relative resistance after deformation divided by the change in deformation. By assuming that the elastomer is not incompressible (i.e., the volume is always constant), the GF can be calculated using (Eq (1)).

(1) $$GF=\left(\frac{\Delta R}{R_{0}\varepsilon_{1}}\right)=\left(\frac{1}{\varepsilon_{1}}\right)\left(\left(\frac{\rho }{\rho_{0}}\right){\left(\varepsilon_{1}+1\right)}^{2}-1\right)$$

Where ΔR is the change of resistance relative to its initial resistance in a certain deformation, R_0_ is the resistance in the unstretched condition, $\varepsilon_{1}$ is the strain of the sensor, ρ is the electrical resistivity of the sensor, and $\rho_{0}$ is the reference electrical resistivity while the sensor is unstretched. The gauge factor was investigated by loading and unloading the sensor from 0 to 200 % strain for ten cycles with a strain rate of 0.1 mm s^−1^. At the end of the loading and unloading step, the sensor was held for 4 s. The effect of the strain rate on the hysteresis and gauge factor was evaluated for the best-performing sample. Three strain rates were employed for the test (i.e., 0.1, 0.5 and 1.0 mm s^−1^). A cyclic test of up to 1500 cycles was also carried out with a strain rate of 0.1 mm s^−1^ and a maximum strain of 60 %. A comparison between loading and unloading the sensor for 0–25 % strain, 0–50 % strain, and 0–100 % strain with a 0.1 mm s^−1^ strain rate was also conducted. The response time of the sensor was also measured by stretching the sensor with a strain rate of 1000 mm s^−1^ into a 5 % strain.

The investigation of the temperature effects on the sensor was also conducted. Three sensors were placed on top of a hot plate stirrer, and the temperature was increased from 25 °C to 75 °C with 5 °C increments. The resistance on each temperature was then measured, and the average of ΔR/R values for each sensor were then calculated.

## 3 Result and discussion

**Fig. 1:**
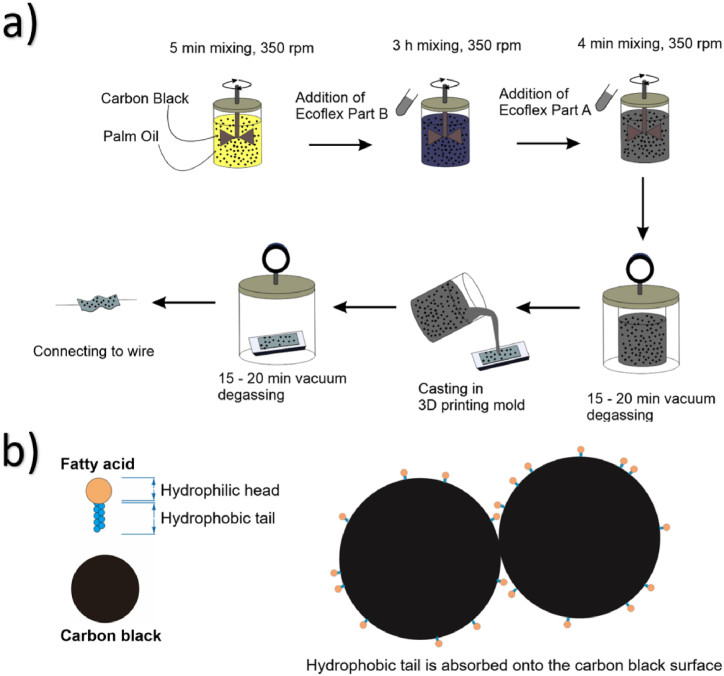
a) The manufacturing of the stretchable piezoresistive strain sensor based on palm oil and carbon black-filled elastomer. b) Illustration for the mechanism of palm oil derived fatty acid in preventing the agglomeration of the carbon black particles.

A schematic of the manufacturing process for the stretchable piezoresistive strain sensor is shown in Fig. 1a. The composition of the carbon black to Ecoflex was set as 12 % since it results in a sensor with the lowest resistance in the literature [1]. The carbon black was first dispersed into the palm oil using a vacuum mixer. The palm oil can successfully disperse the carbon black due to the presence of high palmitic acid that can provide electrostatic repulsion, preventing agglomeration of the carbon black particles [25,29]. The mechanism of palm oil derived fatty acid in preventing the agglomeration of the carbon black particles can be illustrated in Fig. 1b. The hydrophobic tail of the palmitic acid is absorbed on the carbon black surface, and the hydrophilic head induces electrostatic repulsion, preventing agglomeration [29]. Ecoflex 00–30 Part B and Ecoflex 00–30 Part A were then sequentially poured into the mixture, interspersed by the mixing process to ensure the uniformity. A vacuum degassing was applied to eliminate the possibility of the trapped air bubbles. Five stretchable piezoresistive strain sensors with different palm oil compositions were fabricated in this study. The sensors are labelled based on the weight ratio of the palm oil to the total weight of the composite materials (Table 1), i.e., Palm Oil 0.15, Palm Oil 0.21, Palm Oil 0.26, Palm Oil 0.31, and Palm Oil 0.35.

**Fig. 2:**
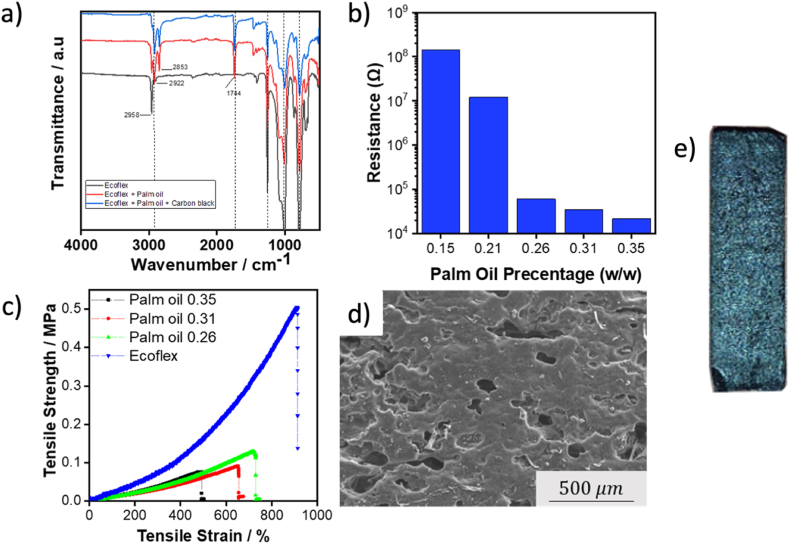
a) Fourier transform infrared (FTIR) spectra of the sensor materials in comparison to Ecoflex and Ecoflex plus palm oil. b) The resistance of piezoresistive strain sensor with various contents of palm oil. c) Tensile test results of the sensors with various palm oil content at the strain rate 100 mm min^−1^. d) Representative scanning electron microscope (SEM) image of the stretchable piezoresistive strain sensor. e) The fabricated sensor with 0.35 palm oil w/w.

FTIR spectra for all the samples were compared with the pure Ecoflex and Ecoflex with the addition of palm oil (Fig. 2a). The FTIR result of Palm Oil 0.35 is given as representative, as all samples show similar patterns of FTIR spectra. FTIR spectra of pure Ecoflex show a good agreement with previous works [31,32]. The peak at 2950 cm^−1^ is attributed to the CH stretching vibrations [31]. The bending asymmetric vibrations of CH are related to the peak at 1400 cm^−1^. Symmetric deformation of Si-CH_3_ and the asymmetric Si-O-Si stretching can be identified from the peaks at 1260 and 1100 cm^−1^ [33]. The addition of palm oil changes the FTIR spectra of the mixture. A small peak at 3008 cm^−1^ is associated with unsaturated fatty acid double bonds [34]. Both peaks at 2922 and 2853 cm^−1^ are obtained from the CH stretching band of the fatty acid hydrocarbon chain, while the peak at 1744 cm^−1^ is related to the ester band C=O stretched [34,35]. Since the additional peaks of the carbon black are mainly derived by carbon bonds, the spectra of Palm Oil 0.35 exhibit a similar pattern to the other two samples [31].

The addition of palm oil changes the resistance of the sensors (Fig. 2b). The higher the weight ratio of the palm oil, the lower the resistance of the sensor, showing a resistance value of 14.3 MΩ, 1.21 MΩ, 60.25 kΩ, 35.12 kΩ, and 21.54 kΩ, for the Palm Oil 0.15, Palm Oil 0.21, Palm Oil 0.26, Palm Oil 0.31, and Palm Oil 0.35, respectively. These resistances are equal to resistivity of 715 kΩ cm, 60.5 kΩ cm, 3.01 kΩ cm, 1.76 kΩ cm, and 1.08 kΩ cm, respsectively. This trend is attributed to the high content of palmitic acid in palm oil, which can increase the dispersion of carbon black [25,29]. In particular, palmitic acid is an amphipathic molecule with a hydrophilic headgroup that can induce electrostatic repulsion among carbon black particles, preventing agglomeration [29]. As a result, the incorporation of palm oil with a high content of palmitic acid (i.e., classified as a long-chain of fatty acids) can increase the dispersity of carbon black, hence improving the sensors conductivity. In particular, long-chain fatty acids are an efficient surface modifier due to their low surface energy and adeptness at chemisorption [36]. Furthermore, the resistance of Palm Oil 0.35 is 60 % lower than that of a sensor prepared with coconut oil solvent with a similar carbon black content [1]. More importantly, the resistances of Palm Oil 0.26, Palm Oil 0.31, and Palm Oil 0.35 sensors are within the requirement for robotic and wearable applications, which is typically in the range of several kiloohms [37], while adding palm oil content of more than 0.35 w/w will reduce its structural integrity.

This work used an average carbon black particle size of 50 nm. Generally, a smaller carbon black size tends to aggregate due to a higher surface area-to-volume ratio, leading to stronger van der Waals forces between them, which promotes agglomeration [38]. Surface treatment, such as the addition of palm oil in this work, can alter the surface properties of the carbon black particle, thereby reducing surface energy and minimizing agglomeration. The impact of surface treatment is typically more pronounced for a smaller particle size [39]. Therefore, we believe that the addition of palm oil will also be useful for a smaller average diameter of carbon black. In elastomer composites, smaller carbon black particles are preferred to produce soft stretchable sensors since it has a lower percolation threshold [40].

The tensile tests were then performed for Palm Oil 0.26, Palm Oil 0.31, and Palm Oil 0.35. Adding palm oil decreases the modulus elasticity and reduces the elongation at break of the sensors (Fig. 2c). Although the modulus elasticity of strain sensors decreases with increasing palm oil, these sensors can still be stretched up to more than 500 %, showing elongation at break of 700 %, 650 %, and 500 % for Palm Oil 0.26, Palm Oil 0.31, and Palm Oil 0.35, respectively. In general, the addition of carbon black results in an increase in the modulus elasticity [22]. The reduction in the modulus elasticity of the sensors observed in this work is related to the use of palm oil, which has the property of a natural plasticizer that would lead to a lower degree of crosslinking within the Ecoflex matrix [41]. In particular, the reduction of modulus elasticity is an advantage in sensor applications since it will not alter the stiffness of the measured system, especially in soft robot applications where a soft material is used [42]. SEM image of the sensor with the lowest resistance (i.e., Palm Oil 0.35) is shown in Fig. 2d. It has a porous structure, which was similar to the previous work using another organic solvent [1]. The porous structure originates from the incorporation of palm oil with a low evaporation rate and persists within the structure throughout the solidification of Ecoflex, resulting in porosity in the final product [1]. The fabricated Palm Oil 0.35 sensor can be seen in Fig. 2e.

**Fig. 3:**
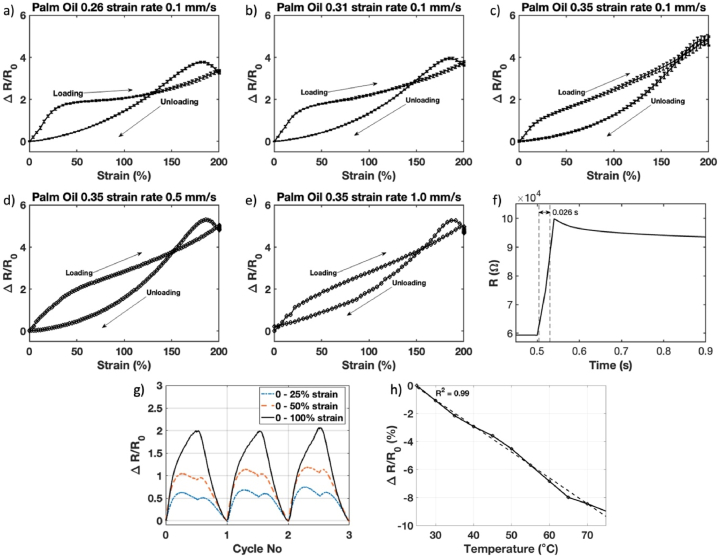
The changes in resistance of the sensor during loading and unloading cycles at 0.1 mm s^−1^ strain rate: a) Palm Oil 0.26, b) Palm Oil 0.31, and c) Palm Oil 0.35. The error bar in a), b), and c) show the standard deviation of the changes in the resistance. The changes in resistance of the Palm Oil 0.35 sensor during loading and unloading cycles under different strain rate: d) 0.5 mm s^−1^ and e) 1.0 mm s^−1^. f) The response time of the sensor while loaded with 5 % strain with a strain rate of 1000 mm s^−1^. The two dashed lines indicate the rise time of the sensor (0.026 s). g) A comparison between the relative change of the sensor's resistance for three cycles with 0–25 % strain, 0–50 % strain, and 0–100 % strain with 0.1 mm s^−1^ strain rate. h) effect of temperatures on the sensor's resistance.

The electromechanical behavior of the sensors under external stress is then investigated. Fig. 3a–c shows the changes in resistance of the sensors with several palm oil compositions while undergoing loading and unloading cycles at a strain rate of 0.1 mm s^−1^. Two strain regions that have different sensitivity can be observed during loading between 0 and 200 % strain. Therefore, the gauge factors were calculated in both regions (i.e., low strain (0–20 %) and high strain (20–200 %) region, Table 2). Palm Oil 0.26 and Palm Oil 0.31 have a high gauge factor in the low strain region, which are 6.12 and 6.00, respectively, while Palm Oil 0.35 has an improved sensitivity in the high strain region with a gauge factor of 2.43.

**Table 2:** Gauge factor and coefficient of determination (*R*^2^) of the palm oil based stretchable piezoresistive strain sensors under various strain rates.

Sample Variance	Strain Rate (mm s^−1^)	Gauge Factor			
		0–20 %	*R*^2^	20–200 %	*R*^2^
Palm Oil 0.26	0.1	6.12	0.99	1.68	0.93
Palm Oil 0.31	0.1	6.00	0.99	1.87	0.98
Palm Oil 0.35	0.1	4.75	0.99	2.43	0.99
Palm Oil 0.35	0.5	5.07	0.99	2.49	0.99
Palm Oil 0.35	1.0	5.18	0.98	2.49	0.99

Furthermore, Palm Oil 0.35 also has better linearity in both strain regions, as can be seen by its coefficient of determination ($R^{2}$) values, which is closer to 1 (Table 2). The higher content of palm oil (and hence palmitic acid) increases the dispersity of the carbon black inside the Ecoflex matrix, which enhances the number of conductive pathways within the sensors [25]. The gauge factor of Palm Oil 0.35 sensor is comparable to similar works that had much lower stretchability (Table 3). In particular, it is higher than the piezoresistive sensor that employed coconut oil as the solvent (i.e., 0.21–0.77) [1].

**Table 3:** Performance comparison of stretchable piezoresistive strain sensor in literature.

No	Materials	Gauge Factor	Hysteresis	Maximum Strain (%)	Reference
1	Ecoflex/CB-palm oil	2.43–4.75	High	500–750	This Study
2	Ecoflex/CB-Coconut Oil	0.21–0.77	High	1000	[1]
3	Ecoflex/CB	1.62–3.37	High	500	[5]
4	Ecoflex/CNT	1.75	Low	500	[6]
5	PDMS/SWCNT-chloroform	N/A	Low	300	[19]
6	PDMS/PTFE-heptane	4–10	High	125	[20]
7	Ecoflex/carbon grease	3.8	High	450	[43]
8	CNT/Plasticized PVC	1.16	Low	100	[44]
9	Ecoflex/CNT	12.43	Low	100	[45]
10	SR/Graphite-Toluene	1.1–5	Low	600	[46]
11	Ecoflex/CNT-IPA solution	26.7	N/A	150	[47]

The error bar in Fig. 3a–c shows the standard deviations from 10 cycles of loading and unloading the sensors, which elucidates high repeatability. We observed that the sensor has a high hysteresis (up to 50 %) between its loading and unloading cycle, which is typical for a piezoresistive strain sensor [1,5,20]. However, the change in resistance is repeatable during the loading and unloading stage. The average standard deviation of the resistances in each position during the loading and unloading cycle is 3 %, which is better than the piezoresistive sensor with heptane solvent (11.2 %) [1]. The effect of strain rate on the sensitivity of the sensors was also investigated for the best-performing sensor (i.e., Palm Oil 0.35). Fig. 3c–e shows the sensor has a stable response for up to 1.0 mm s^−1^ strain rate. In the 0–20 % strain region, its gauge factors at 0.5 and 1.0 mm s^−1^ strain rates are higher than those at 0.1 mm s^−1^ (Table 2), which is related to the sudden movements during fast deformations that may disturb the conductive paths [17,48]. After enough time, the conductive filler will have adequate time to adjust its position until it reaches a steady state, which occurs at the 20–200 % strain regions.

The bandwidth of the sensor was also investigated by measuring the response time of the sensor while being stretched into a 5 % strain at a strain rate of 1000 mm s^−1^. As can be seen in Fig. 3f, the sensor has a fast response time, which is about 0.026 s. By assuming a first-order system, the bandwidth of the sensor with 0.026 s rise time was 13 Hz. This bandwidth shows that the sensor is suitable for soft robotics and biomechanics applications [1,48].

A comparison between the relative changes in the sensor's resistance during the loading and unloading cycle for different maximum strain values (i.e. 25 %, 50 %, and 100 %) can be seen in Fig. 3g, which follows the work in the literature [[49], [50], [51]]. During the loading and unloading cycles, the sensors follow similar measurement values. However, it can be seen that the overshoot is much higher for a smaller maximum strain value (i.e. 25 % strain). All of the measurements have the same strain rate of 0.1 mm s^−1^. Therefore, the higher maximum strain values have more time for the percolation network to stabilize, hence a smaller overshoot.

The effect of temperature on changes in the sensor's resistance in the temperature range of 25 °C–75 °C was also investigated. Three sensors were tested, and the mean values of the change in their resistance values are shown in Fig. 3h. As shown in Fig. 3h, the resistance of the sensor decreases with the increase in temperature, which is typical for a stretchable piezoresistive sensor [1]. The maximum change in ΔR/R is 8.9 % ± 3.4 %, which is observed at a temperature of 75 °C. This value is comparable with another stretchable piezoresistive sensor in the literature [5]. Notably, the resistance changes are linear ($R^{2}$ = 0.99), with a decrease of 0.18 % per 1 °C temperature increment.

**Fig. 4:**
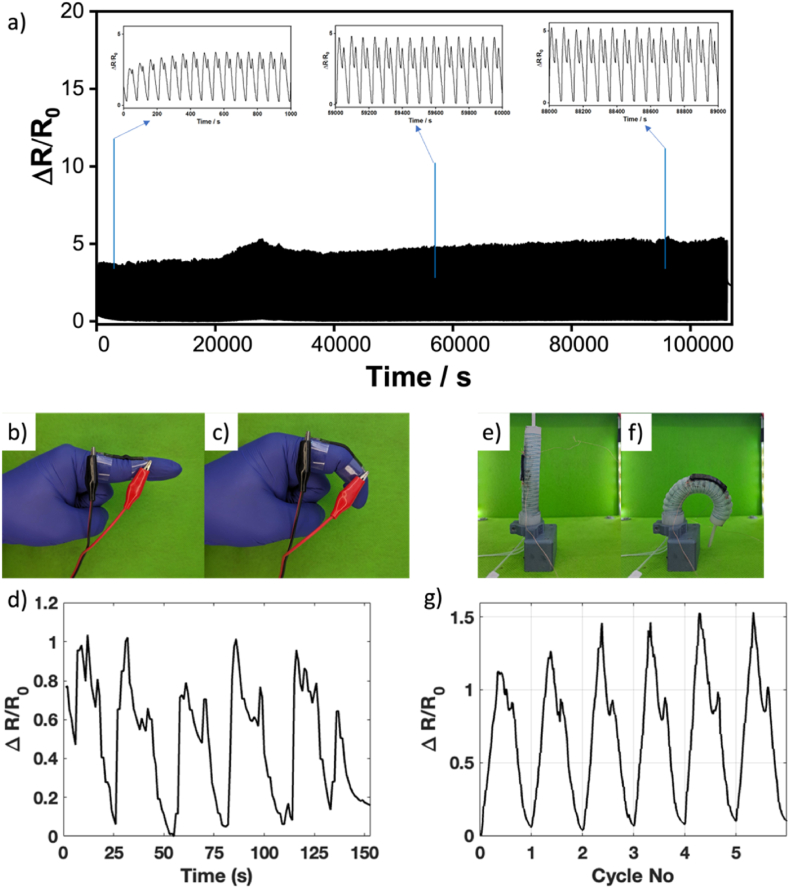
a) Relative change of the piezoresistive strain sensor resistance after 1500 loading-unloading cycling test. Photographs of the piezoresistive strain sensor prototype during the finger bending measurement: b) in the straight position, c) during bending. d) The bending test result of the piezoresistive strain sensor prototype during the finger bending measurement. Photographs of the piezoresistive strain sensor prototype for measuring bending a pneumatic soft robot. e) in the straight position (0 kPa pressure), f) during bending (75 kPa pressure). g) The test result of the piezoresistive strain sensor prototype during the soft robot bending measurement.

Repeatability is also important when strain sensors are used as wearables, which are subjected to repeated stretch and release cycles. Fig. 4a shows the change of the resistance value over 1500 cycles of stretching and releasing at 60 % tension. The resistance of the sensor increases during stretching and decreases during relaxation. It becomes stable after a few cycles and exhibits good repeatability. The stability can be estimated by the standard deviation of the resistance at the 0 % strain (R_0_) and 60 % strain (R_60_), showing the R_0_ and the R_60_ at 4 % and 5 % after 1500 cycles, respectively. Before maintaining a steady trend, the strain response tends to go down in the first few cycles, which is expected from strain sensors and can be attributed to dynamic changes in the conductive path within the sensor [5,8,44], resulting from the random distribution of nanofiller within the matrix. Generally, the more cycles applied, the greater the observed change in resistance, a trend that aligns with findings in the literature [52]. The random distribution of nanofillers is essential to maintain conductivity even under mechanical deformation. However, in certain instances, such as at the 20,000^th^ and 40,000^th^ seconds in Fig. 4a, this randomness can lead to sudden fluctuations in resistance values. During these times, the lowest resistance values were higher than in other cycles. With additional cycles, the trend gradually stabilized, as observed in the subsequent cycles.

The dynamic changes in the conductive path can also be observed with the overshoot of the resistance value at the end of the stretching cycle and at the beginning of the releasing cycle. The conductivity of soft stretchable sensors arises from the formation of percolation networks created by nanofillers embedded within the matrix. During rapid strain changes, these conductive pathways can be disrupted, leading to fluctuations in electrical conductivity and causing an overshoot in sensor readings, as observed during both the stretching and releasing cycles [53]. Hence, the two peaks are observed during one stretching and releasing cycle. The appearance of these dual peaks has also been documented in the literature [43].

As proof of concept, the palm oil-based piezoresistive strain sensor was employed to detect the joint motion of the human body (e.g., finger bending). In principle, the proof-of-concept testing for the strain sensor in this study followed the methodology of previous work, in which the sensor was securely attached to a human body to reliably detect real-time movement changes [[49], [50], [51]]. The sensor resistances were recorded during the bending-straightening motions of the finger to around a 60° angle (Fig. 4b–d). During bending, the sensor undergoes stretching that increases its resistance, showing a gradual increase in the ΔR/R_0_ value (Fig. 4d). The strain sensor is then returned to its unloading state as soon as the finger joint is straightened, causing the ΔR/R_0_ to reset. Specifically, the bending and straightening motions of the finger are clearly distinguishable through the observed resistance changes, demonstrating the strain sensor's capability to detect real-time movement [50].

In addition, a palm oil-based piezoresistive strain sensor was deployed to measure the bending of a pneumatic soft robot [54]. The pressure was manually changed from 0 to 75 kPa during pressurization stage and changed back from 75 to 0 kPa during depressurization stage. The soft robot was in a straight position (Fig. 4e) with a 0 kPa pressure and at approximately 200° angle with a pressure of 0.75 kPa (Fig. 4f). As can be seen in Fig. 4g, during bending, the sensor stretches that increased its resistance. The strain sensor is then returned to its unloading state as soon as the robot is depressurized, causing the robot to return to its straight state. During the experiment, the pressure was adjusted manually. Hence, there is a difference in the observed maximum peak on each cycle.

## 4 Conclusion

In summary, an affordable and stretchable piezoresistive strain sensor was successfully fabricated using palm oil as the biocompatible organic solvent. The use of palm oil, which has high palmitic acid content, enhances the dispersibility of the carbon black in the elastomer matrix, consequently leading to a significant improvement in sensor conductivity. Palm oil also acts as a plasticizer; hence, it can reduce the degree of crosslinking within the matrix and enable the production of highly stretchable composite materials with low modulus elasticity. In this work, incorporating palm oil as the additive solvent in elastomer (EcoFlex 00–30) can provide a stretchable piezoresistive sensor with better electrical conductivity and better linearity compared to a sensor without any solvent. We found that the optimum palm oil composition in the mixture (i.e., 0.35 w/w) could result in a high stretching performance of up to 500 % strain and a low resistance value of 21.54 kΩ. Moreover, the strain sensor shows remarkable reversibility (over 1500 cycles at 60 % without deteriorating in its performance), a fast rise time of 0.026 s, and a gauge factor of more than 2. Using a cheap and abundant palm oil-based organic solvent has the potential to be a promising additive in the synthesis of stretchable piezoresistive sensors. The stretchable piezoresistive strain sensor that was produced in this work is suitable for detecting human body movements and soft robotics applications.

## CRediT authorship contribution statement

**Vani Virdyawan:** Writing – review & editing, Writing – original draft, Visualization, Supervision, Project administration, Methodology, Investigation, Funding acquisition, Formal analysis, Data curation, Conceptualization. **Thoriq Marendra:** Writing – review & editing, Writing – original draft, Visualization, Investigation, Formal analysis, Data curation. **Bagas Prakoso:** Writing – review & editing, Writing – original draft, Visualization, Investigation, Formal analysis, Data curation. **Indrawanto:** Writing – review & editing, Validation, Supervision, Project administration, Methodology, Funding acquisition, Conceptualization, Writing – review & editing, Validation, Supervision, Project administration, Methodology, Funding acquisition, Conceptualization. **Afriyanti Sumboja:** Writing – review & editing, Writing – original draft, Validation, Supervision, Project administration, Methodology, Funding acquisition, Conceptualization.

## Data availability

Data will be made available on request.

## Declaration of competing interest

The authors declare that they have no known competing financial interests or personal relationships that could have appeared to influence the work reported in this paper.
